# 2-Amino-4,5,6,7-tetra­hydro­benzo[*b*]thio­phene-3-carbonitrile

**DOI:** 10.1107/S1600536811045338

**Published:** 2011-11-02

**Authors:** Willams L. Silva, Maria do Carmo A. de Lima, Suely L. Galdino, Ivan R. Pitta, Carlos A. De Simone

**Affiliations:** a Laboratório de Síntese e Vetorização de Moléculas, Bioativas., Universidade Estadual da Paraíba, 58020-540 João Pessoa, PB, Brazil; b Laboratório de Síntese e Planejamento de Fármacos, Departamento de Antibióticos, Universidade Federal de Pernambuco, 50670-910 Recife, PE, Brazil; c Departamento de Física e Informática, Instituto de Física de São Carlos, Universidade de São Paulo - USP, 13560-970 São Carlos, SP, Brazil

## Abstract

The title compound, C_9_H_10_N_2_S, was synthesized according to Gewald procedures by the reaction of cyclo­hexa­none with malonitrile and sulfur in the presence morpholine. The cyclo­hexane ring adopts a half-chair conformation and the thio­phene ring is essentially planar (r.m.s. deviation = 0.05 Å). The crystal packing is stabilized by two inter­molecular N—H⋯N hydrogen bonds, which link the mol­ecules into centrosymmetric rings with graph-set motif *R*_2_^2^(12).

## Related literature

For background to 2-amino thio­phenes, see: Puterová *et al.* (2009 ▶). For anti­arrhythmic and serotonin antagonist properties of 2-substituted thio­phene derivatives, see: Amr *et al.* (2010 ▶). For their analgesic or anti-inflammatory activity, see: Hafez & El-Gazzar (2008 ▶). For the synthesis of 2-amino thio­phenes, see: Gewald *et al.* (1966 ▶); Wang *et al.* (2010 ▶). For similar structures, see: Larson & Simonsen (1988 ▶); Mendonça Junior *et al.* (2010 ▶). For puckering parameters, see: Cremer & Pople (1975 ▶).  For hydrogen-bond motifs, see: Bernstein *et al.* (1995 ▶).
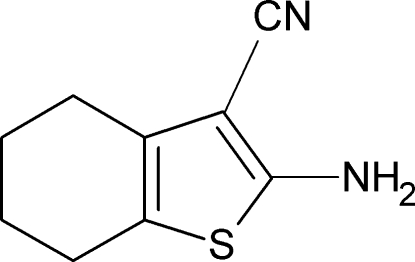


## Experimental

### 

#### Crystal data

C_9_H_10_N_2_S
*M* *_r_* = 178.25
Monoclinic, 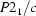

*a* = 10.4274 (3) Å
*b* = 8.1487 (3) Å
*c* = 13.2342 (4) Å
β = 126.937 (2)°
*V* = 898.81 (5) Å^3^
*Z* = 4
Mo *K*α radiation
μ = 0.30 mm^−1^
*T* = 295 K
0.22 × 0.22 × 0.20 mm

#### Data collection

Nonius KappaCCD diffractometer
12462 measured reflections
2058 independent reflections
1630 reflections with *I* > 2σ(*I*)
*R*_int_ = 0.052

#### Refinement

*R*[*F* ^2^ > 2σ(*F* ^2^)] = 0.045
*wR*(*F* ^2^) = 0.137
*S* = 1.04
2058 reflections
109 parameters
H-atom parameters constrained
Δρ_max_ = 0.19 e Å^−3^
Δρ_min_ = −0.36 e Å^−3^

### 

Data collection: *COLLECT* (Nonius, 1997 ▶); cell refinement: *SCALEPACK* (Otwinowski & Minor, 1997 ▶); data reduction: *DENZO* (Otwinowski & Minor, 1997 ▶) and *SCALEPACK*; program(s) used to solve structure: *SHELXS97* (Sheldrick, 2008 ▶); program(s) used to refine structure: *SHELXL97* (Sheldrick, 2008 ▶); molecular graphics: *ORTEP-3 for Windows* (Farrugia, 1997 ▶); software used to prepare material for publication: *WinGX* (Farrugia, 1999 ▶).

## Supplementary Material

Crystal structure: contains datablock(s) I, global. DOI: 10.1107/S1600536811045338/bx2379sup1.cif

Structure factors: contains datablock(s) I. DOI: 10.1107/S1600536811045338/bx2379Isup2.hkl

Supplementary material file. DOI: 10.1107/S1600536811045338/bx2379Isup3.cml

Additional supplementary materials:  crystallographic information; 3D view; checkCIF report

## Figures and Tables

**Table 1:** Hydrogen-bond geometry (Å, °)

*D*—H⋯*A*	*D*—H	H⋯*A*	*D*⋯*A*	*D*—H⋯*A*
N2—H2*A*⋯N1^i^	0.86	2.28	3.121 (2)	166
N2—H2*B*⋯N1^ii^	0.86	2.42	3.225 (3)	155

Symmetry codes: (i) 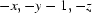; (ii) 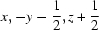.

**Fig. 1.**
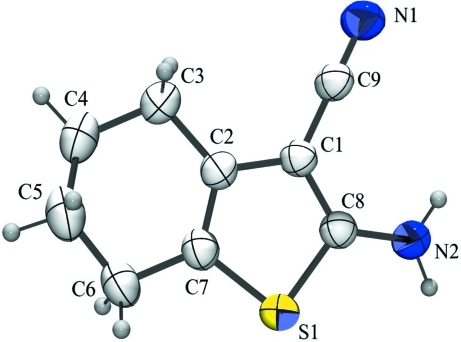
The structure of (I), showing the atom-labelling scheme. Displacement ellipsoids are shown at the 50% probability level.

**Fig. 2.**
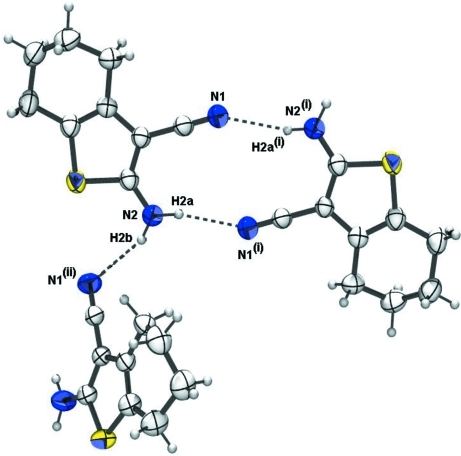
Part of the crystal structure showing the formation of centrosymmetric dimers R22(12) rings. [Symmetry codes: (i) -x, -y-1, -z; (ii) x, -y-1/2, z+1/2.]

## supplementary crystallographic information

### Comment

Thiophenes and their fused heterocyclic ring systems possess a wide spectrum of biological activities, as antiarrhythmic and serotonin antagonist (Amr *et al.*, 2010) and analgesic or anti-inflammatory activities (Hafez & El-Gazzar, 2008). In this work, the title compound was obtained according Gewald procedures, by the reaction of cyclohexanone with malonitrile and sulfur in the presence morpholine (Gewald *et al.*, 1966; Wang *et al.*, 2010). In the title compound, the cyclohexane ring adopts a half-chair conformation with calculated puckering parameters of:  Q_T_= 0.487 (1) Å, θ = 50.6 (1)°, φ = 148.6 (2)° (Cremer & Pople, 1975). The crystal packing is stabilized by two intermolecular N—H···N  hydrogen bonds, which links the molecules into rings with graph-set notation R_2_^2^(12), Table 1 & Fig.2.

### Experimental

Crystals suitable for single-crystal X-ray diffraction were grown by slow evaporation at room temperature of a solution from ethanol/water.

### Refinement

All H atoms attached were fixed geometrically and treated as riding with C—H = 0.97 Å (methylene) and N—H = 0.86 Å and with *U*_iso_(H) = 1.2*U*_eq_(C or N).

### Crystal data

**Table:**

C_9_H_10_N_2_S	*F*(000) = 376
*M**_r_* = 178.25	*D*_x_ = 1.317 Mg m^−^^3^
Monoclinic, *P*2_1_/*c*	Mo *K*α radiation, λ = 0.71073 Å
Hall symbol: -P 2ybc	Cell parameters from 6505 reflections
*a* = 10.4274 (3) Å	θ = 2.6–27.5°
*b* = 8.1487 (3) Å	µ = 0.30 mm^−^^1^
*c* = 13.2342 (4) Å	*T* = 295 K
β = 126.937 (2)°	Prism, yellow
*V* = 898.81 (5) Å^3^	0.22 × 0.22 × 0.20 mm
*Z* = 4	

### Data collection

**Table:**

Nonius KappaCCD diffractometer	1630 reflections with *I* > 2σ(*I*)
Radiation source: Enraf–Nonius FR590	*R*_int_ = 0.052
horizonally mounted graphite crystal	θ_max_ = 27.5°, θ_min_ = 3.1°
Detector resolution: 9 pixels mm^-1^	*h* = −13→13
CCD rotation images,thick slices scans	*k* = −10→10
12462 measured reflections	*l* = −17→17
2058 independent reflections	

### Refinement

**Table:**

Refinement on *F*^2^	Primary atom site location: structure-invariant direct methods
Least-squares matrix: full	Secondary atom site location: difference Fourier map
*R*[*F*^2^ > 2σ(*F*^2^)] = 0.045	Hydrogen site location: inferred from neighbouring sites
*wR*(*F*^2^) = 0.137	H-atom parameters constrained
*S* = 1.04	*w* = 1/[σ^2^(*F*_o_^2^) + (0.1*P*)^2^] where *P* = (*F*_o_^2^ + 2*F*_c_^2^)/3
2058 reflections	(Δ/σ)_max_ = 0.001
109 parameters	Δρ_max_ = 0.19 e Å^−^^3^
0 restraints	Δρ_min_ = −0.36 e Å^−^^3^

### Special details

**Table:**

Geometry. All e.s.d.'s (except the e.s.d. in the dihedral angle between two l.s. planes) are estimated using the full covariance matrix. The cell e.s.d.'s are taken into account individually in the estimation of e.s.d.'s in distances, angles and torsion angles; correlations between e.s.d.'s in cell parameters are only used when they are defined by crystal symmetry. An approximate (isotropic) treatment of cell e.s.d.'s is used for estimating e.s.d.'s involving l.s. planes.
Refinement. Refinement of *F*^2^ against ALL reflections. The weighted *R*-factor *wR* and goodness of fit *S* are based on *F*^2^, conventional *R*-factors *R* are based on *F*, with *F* set to zero for negative *F*^2^. The threshold expression of *F*^2^ > σ(*F*^2^) is used only for calculating *R*-factors(gt) *etc*. and is not relevant to the choice of reflections for refinement. *R*-factors based on *F*^2^ are statistically about twice as large as those based on *F*, and *R*- factors based on ALL data will be even larger.

### Fractional atomic coordinates and isotropic or equivalent isotropic displacement parameters (Å^2^)

**Table:**

	*x*	*y*	*z*	*U*_iso_*/*U*_eq_	
S1	0.22127 (5)	0.01327 (5)	0.27769 (4)	0.0543 (2)	
N1	0.0577 (2)	−0.37778 (18)	−0.07760 (14)	0.0648 (4)	
N2	0.07860 (18)	−0.28276 (18)	0.20995 (14)	0.0603 (4)	
H2A	0.0428	−0.3698	0.1640	0.072*	
H2B	0.0719	−0.2727	0.2713	0.072*	
C1	0.16686 (17)	−0.15535 (17)	0.09245 (13)	0.0426 (3)	
C2	0.24712 (19)	−0.00893 (18)	0.09686 (15)	0.0445 (4)	
C3	0.2917 (2)	0.0288 (2)	0.01054 (17)	0.0532 (4)	
H3A	0.1965	0.0603	−0.0720	0.064*	
H3B	0.3365	−0.0684	0.0002	0.064*	
C4	0.4146 (2)	0.1681 (2)	0.06489 (19)	0.0662 (5)	
H4A	0.5188	0.1265	0.1345	0.079*	
H4B	0.4235	0.2079	0.0003	0.079*	
C5	0.3678 (2)	0.3083 (2)	0.1112 (2)	0.0690 (5)	
H5A	0.2627	0.3485	0.0419	0.083*	
H5B	0.4439	0.3974	0.1389	0.083*	
C6	0.3640 (2)	0.2565 (2)	0.22012 (18)	0.0641 (5)	
H6A	0.4725	0.2486	0.2975	0.077*	
H6B	0.3068	0.3380	0.2325	0.077*	
C7	0.28162 (17)	0.0932 (2)	0.19015 (15)	0.0499 (4)	
C8	0.14543 (17)	−0.16192 (19)	0.18582 (13)	0.0451 (4)	
C9	0.10751 (18)	−0.27940 (19)	−0.00076 (14)	0.0477 (4)	

### Atomic displacement parameters (Å^2^)

**Table:**

	*U*^11^	*U*^22^	*U*^33^	*U*^12^	*U*^13^	*U*^23^
S1	0.0624 (3)	0.0552 (3)	0.0567 (3)	−0.01030 (17)	0.0419 (3)	−0.01544 (17)
N1	0.0984 (12)	0.0474 (8)	0.0663 (9)	−0.0081 (8)	0.0589 (9)	−0.0091 (7)
N2	0.0834 (10)	0.0550 (9)	0.0623 (8)	−0.0155 (7)	0.0543 (8)	−0.0106 (6)
C1	0.0474 (7)	0.0386 (7)	0.0442 (7)	0.0030 (6)	0.0288 (6)	−0.0003 (6)
C2	0.0430 (8)	0.0457 (8)	0.0457 (8)	0.0023 (6)	0.0271 (7)	0.0008 (6)
C3	0.0579 (10)	0.0550 (10)	0.0544 (9)	0.0025 (7)	0.0378 (8)	0.0051 (7)
C4	0.0629 (10)	0.0718 (13)	0.0748 (11)	−0.0065 (9)	0.0472 (9)	0.0060 (9)
C5	0.0694 (11)	0.0575 (11)	0.0780 (12)	−0.0141 (9)	0.0432 (10)	−0.0016 (9)
C6	0.0630 (10)	0.0561 (10)	0.0719 (11)	−0.0175 (8)	0.0398 (9)	−0.0164 (8)
C7	0.0469 (8)	0.0497 (9)	0.0539 (8)	−0.0047 (6)	0.0308 (7)	−0.0056 (7)
C8	0.0466 (7)	0.0441 (8)	0.0468 (8)	0.0014 (6)	0.0292 (7)	−0.0020 (6)
C9	0.0618 (9)	0.0409 (8)	0.0500 (8)	0.0028 (6)	0.0387 (7)	0.0026 (6)

### Geometric parameters (Å, °)

**Table:**

S1—C8	1.7280 (15)	C3—H3A	0.9700
S1—C7	1.7432 (17)	C3—H3B	0.9700
N1—C9	1.145 (2)	C4—C5	1.509 (3)
N2—C8	1.351 (2)	C4—H4A	0.9700
N2—H2A	0.8600	C4—H4B	0.9700
N2—H2B	0.8600	C5—C6	1.526 (3)
C1—C8	1.383 (2)	C5—H5A	0.9700
C1—C9	1.417 (2)	C5—H5B	0.9700
C1—C2	1.438 (2)	C6—C7	1.502 (2)
C2—C7	1.346 (2)	C6—H6A	0.9700
C2—C3	1.502 (2)	C6—H6B	0.9700
C3—C4	1.530 (2)		
			
C8—S1—C7	92.09 (7)	H4A—C4—H4B	108.0
C8—N2—H2A	120.0	C4—C5—C6	111.88 (16)
C8—N2—H2B	120.0	C4—C5—H5A	109.2
H2A—N2—H2B	120.0	C6—C5—H5A	109.2
C8—C1—C9	121.99 (14)	C4—C5—H5B	109.2
C8—C1—C2	113.48 (13)	C6—C5—H5B	109.2
C9—C1—C2	124.47 (13)	H5A—C5—H5B	107.9
C7—C2—C1	112.23 (14)	C7—C6—C5	109.33 (15)
C7—C2—C3	122.47 (14)	C7—C6—H6A	109.8
C1—C2—C3	125.29 (14)	C5—C6—H6A	109.8
C2—C3—C4	110.43 (14)	C7—C6—H6B	109.8
C2—C3—H3A	109.6	C5—C6—H6B	109.8
C4—C3—H3A	109.6	H6A—C6—H6B	108.3
C2—C3—H3B	109.6	C2—C7—C6	125.82 (15)
C4—C3—H3B	109.6	C2—C7—S1	111.97 (12)
H3A—C3—H3B	108.1	C6—C7—S1	122.20 (13)
C5—C4—C3	111.65 (15)	N2—C8—C1	128.53 (14)
C5—C4—H4A	109.3	N2—C8—S1	121.26 (11)
C3—C4—H4A	109.3	C1—C8—S1	110.21 (11)
C5—C4—H4B	109.3	N1—C9—C1	178.84 (18)
C3—C4—H4B	109.3		

### Hydrogen-bond geometry (Å, °)

**Table:**

*D*—H···*A*	*D*—H	H···*A*	*D*···*A*	*D*—H···*A*
N2—H2A···N1^i^	0.86	2.28	3.121 (2)	166
N2—H2B···N1^ii^	0.86	2.42	3.225 (3)	155

Symmetry codes: (i) −*x*, −*y*−1, −*z*; (ii) *x*, −*y*−1/2, *z*+1/2.
